# Valorization of Mango Peel Powder in Spreadable Cream Cheese: Effects on Composition, Bioactive Properties, Structure, and Sensory Quality

**DOI:** 10.3390/foods15203689

**Published:** 2026-10-16

**Authors:** Ionuț Dumitru Veleșcu, Florina Stoica, Ioana Cristina Crivei, Andreea Bianca Balint, Roxana Nicoleta Rațu

**Affiliations:** 1 Department of Food Technologies, Faculty of Agriculture, “Ion Ionescu de la Brad” Iasi University of Life Sciences, 3 Mihail Sadoveanu Alley, 700489 Iasi, Romania; ionut.velescu@iuls.ro (I.D.V.); ioana.crivei@iuls.ro (I.C.C.); bianca.balint@iuls.ro (A.B.B.); 2 Research Institute for Agriculture and Environment, “Ion Ionescu de la Brad” Iasi University of Life Sciences, 14 Sadoveanu Alley, 700490 Iasi, Romania; florina.stoica@iuls.ro

**Keywords:** fruit-processing by-products, dietary fiber, phenolic compounds, carotenoids, antioxidant activity, dairy fortification, scanning electron microscopy, circular food systems

## Abstract

This study characterized mango peel powder (MPP) and evaluated its effects on the composition, bioactive properties, structure, and sensory quality of spreadable cream cheese. Three formulations were prepared, namely an unsupplemented control and two supplemented cheeses, designated MPPC1 and MPPC2, with nominal MPP addition levels of 3 and 6 g/100 g of the curd–butter mixture before powder addition, respectively. Physicochemical composition, phytochemical content, antioxidant activity using the 2,2′-azino-bis(3-ethylbenzothiazoline-6-sulfonic acid)(ABTS) assay, colour, texture, microstructure, and sensory properties were evaluated. Phytochemical content, ABTS activity, and colour were monitored during 21 days of refrigerated storage, whereas the other assessments were performed at day 0. Moisture decreased from 60.72% in the control to 55.69% and 51.97% in MPPC1 and MPPC2, while crude fiber increased from 0.00% in the control to 1.54% and 3.05% in MPPC1 and MPPC2, respectively (*p* < 0.05). Supplemented cheeses showed higher flavonoid and polyphenol contents and ABTS activity than the control. Carotenoids were measured only in the supplemented formulations, with MPPC2 showing the highest concentration. ABTS activity retention ranged from 99.50% to 99.64% after storage. Increasing MPP addition produced darker, redder, yellower, and firmer cheeses; hardness increased from 4.38 N to 5.59 N and 7.09 N (*p* < 0.05). Microscopy revealed greater structural heterogeneity at higher addition levels. In the preliminary sensory evaluation, MPPC1 achieved the highest overall acceptability score (6.81/7), significantly exceeding the control and MPPC2 (*p* < 0.05). Among the tested formulations, MPPC1 combined compositional enrichment with the highest sensory acceptability, while MPPC2 produced greater phytochemical enrichment and more pronounced colour and texture changes.

## 1. Introduction

Cream cheese is a complex semi-solid dairy system whose quality depends on the composition and interactions of its protein, fat, and aqueous phases. Ingredient composition, particle dispersion, rheological behaviour, texture, flavour release, oxidative stability, and storage stability all influence product performance, while spreadability, mouthfeel, colour, and flavour remain key attributes for consumer acceptance [1,2]. Therefore, the development of nutritionally improved spreadable dairy products requires a careful balance between compositional enhancement and preservation of the sensory and structural characteristics expected for this product category [3].

In recent years, consumer interest in foods formulated with recognizable and minimally processed ingredients has encouraged the use of plant-derived materials as alternatives to conventional colourants, flavouring agents, and texturizing ingredients. Although “clean label” has no universally accepted regulatory definition, it is commonly associated with perceived naturalness, shorter ingredient lists, and reduced use of unfamiliar additives. Fruit-derived powders are particularly attractive in this context because they can provide dietary fiber, pigments, organic acids, flavour compounds, soluble solids, and phytochemicals, while also modifying the technological properties of the food matrix [3].

Mango (*Mangifera indica* L.) is one of the major tropical fruits produced worldwide, with annual production exceeding 55 million tonnes, particularly in countries such as India, China, and Indonesia [4]. The demand for mango-derived products, including juices, purées, nectars, canned products, and dried products, is expected to increase further [5]. However, mango processing generates considerable amounts of peel and seed kernel residues. Mango peel may represent approximately 15–20% of the fruit mass, while the total proportion of residues can be higher depending on the processing method [6]. These by-products are often discarded, used in low-value applications, or allowed to decompose, despite their content of valuable food constituents [7,8]. Their recovery as functional ingredients is therefore consistent with circular-economy strategies aimed at reducing food-processing waste and reintegrating valuable compounds into the food chain [9].

The composition of mango-derived materials depends on cultivar, maturity, and processing conditions. Mango fruit contains carbohydrates, organic acids, vitamins, minerals, volatile compounds, carotenoids, and phenolic compounds, while drying can improve ingredient stability and facilitate incorporation into food matrices. However, drying conditions may influence phytochemical retention and antioxidant activity, as shown by studies on mango pulp, peel, and mango-derived powders [10,11]. Mango peel is especially relevant because it differs substantially from the edible pulp, containing dietary fiber, pectin, carotenoids, minerals, and phenolic compounds such as mangiferin, gallic acid, quercetin, and chlorogenic acid [12,13,14]. Pectin-derived oligosaccharides from mango peel have also been investigated for potential interactions with intestinal microbiota, and the peel has been reported to contain relevant mineral elements [15,16]. Drying and milling can therefore convert this perishable residue into mango peel powder (MPP), a stable ingredient suitable for food applications [17,18]. Nevertheless, the allergenic potential of mango peel should be considered, as alk(en)ylresorcinols have been associated with contact dermatitis, and their concentrations may vary with cultivar and processing conditions [19,20].

The technological applicability of MPP has been studied in several food matrices. In biscuits and cookies, moderate mango peel incorporation increased dietary fiber and antioxidant properties while maintaining acceptable sensory characteristics [21,22]. Mango peel flour has also been used in bread and other bakery products, improving fiber and phenolic content, although effects on technological quality were formulation-dependent [23,24]. Encapsulated mango peel components have been incorporated into noodles as carriers of phenolic compounds and carotenoids [25], while mango and other fruit or vegetable peel flours have also been evaluated as bioactive ingredients in fermented dairy systems [26]. These studies indicate that mango-processing residues can provide both nutritionally active compounds and structurally relevant plant constituents.

Dairy matrices are suitable systems for this type of valorization because products such as cream cheese naturally contain little or no dietary fiber and limited amounts of plant-derived phytochemicals. The incorporation of fruit and vegetable by-products may therefore improve nutritional composition while modifying structure and sensory quality. Ingredients derived from grape pomace, tomato peel, broccoli, artichoke, and corn bran have been investigated in cheese matrices [27], while curry leaf powder [28], Citrus junos peel powder [29], and orange peel-derived ingredients in kefir-based cream cheese [30] have also been studied. These ingredients can affect pH, colour, antioxidant properties, rheological behaviour, microbiological characteristics, and sensory acceptance, depending on their composition and incorporation level. Cream cheese is particularly relevant for such studies because its acceptability depends on a smooth, homogeneous structure and balanced flavour profile [2,31]. Mango peel may contribute carotenoids, phenolics, dietary fiber, and other constituents, but excessive incorporation can alter colour, firmness, smoothness, homogeneity, and flavour, potentially increasing grittiness or reducing sensory quality [32,33,34].

For experimental dairy products, formulation studies must also be conducted under controlled processing and handling conditions because raw materials, processing temperature, ingredient dosing, sanitation, and batch identification can influence product quality and analytical reproducibility. Thus, appropriate raw-material control, processing records, and traceability remain important elements of experimental product development [35,36,37,38].

Accordingly, the present study investigated the incorporation of MPP into spreadable cream cheese at two supplementation levels, 3% and 6%, compared with an unsupplemented control. The formulations were evaluated for physicochemical composition, phytochemical content, antioxidant activity, mineral profile, instrumental colour, texture, and microstructural characteristics. Changes in phytochemical compounds and antioxidant activity were monitored during 21 days of refrigerated storage, and sensory evaluation was used to assess the effect of MPP concentration on product perception and overall acceptability. By integrating compositional, physicochemical, structural, and sensory measurements, the study aimed to determine how increasing MPP incorporation modifies the cream cheese matrix and to identify the supplementation level that provides the most favourable balance between nutritional enhancement and product quality.

## 2. Materials and Methods

### 2.1. Reagents and Chemicals

Analytical-grade reagents and solvents, including gallic acid, catechin, Trolox, ABTS, the Folin–Ciocâlteu reagent, aluminium chloride, sodium nitrite, sodium hydroxide, potassium persulfate, ethanol, hexane, and acetone, were obtained from Sigma-Aldrich (Steinheim, Germany).

### 2.2. Preparation of Mango Peel Powder

Mangoes (*Mangifera indica* L., cv. Chaunsa) were purchased from a local market in Iași, Romania. The fruits were washed and peeled manually. The peels were rinsed with warm water, dried at 40 °C in a dehydrator (GGM Gastro, Chitila, Romania), ground with a Panasonic MX-AC400 mixer grinder (Panasonic Appliances India Company Limited, Chennai, India), and sieved to a particle size of 200–250 µm. The sieved MPP was spread in a thin layer and exposed to a pulsed-light treatment before storage and incorporation into the cheese. The treatment was intended to reduce the microbial load of the powder. The resulting mango peel powder (MPP) was stored in light-protected glass containers at 18 °C in the dark until analysis and cheese preparation.

### 2.3. Extraction and Characterization of MPP

Phytochemicals were extracted using a modified ultrasound-assisted procedure [39]. For polyphenol and flavonoid analyses, 1 g of MPP was mixed with 10 mL of 70% ethanol. Carotenoids were extracted separately from 1 g of MPP using 10 mL of hexane/acetone (3:1, *v*/*v*). Both mixtures were sonicated for 30 min at 30 °C in an Elmasonic S 180 H bath (Elma Schmidbauer GmbH, Singen, Germany); 40 kHz, nominal power 100 W.

Total carotenoids were measured at 450 nm using a Specord 210 Plus UV–Vis spectrophotometer (Analytik Jena, Jena, Germany) and expressed as mg/g dry weight (dw) [40]. Total polyphenols were determined by the Folin–Ciocâlteu method at 765 nm and expressed as mg gallic acid equivalents (GAE)/g dw [41]. Total flavonoids were determined by the aluminium chloride method at 510 nm and expressed as mg catechin equivalents (CE)/g dw [42].

Antioxidant activity was determined using the ABTS radical cation assay [43]. A solution containing 7 mM ABTS and 2.45 mM potassium persulfate was kept in the dark for 16 h. Sample extract (200 µL) was mixed with 1800 µL of the ABTS working solution; after 2 h in the dark, absorbance was measured at 734 nm. Results were expressed as µmol Trolox equivalents (TE)/g dw and as percentage inhibition. Moisture, ash, protein, lipid, crude fiber, and carbohydrate contents were determined as described in [41,44] and expressed as percentages of powder mass.

MPP colour was measured with a CR-410 portable colorimeter (Konica Minolta Inc., Tokyo, Japan) using d/0° geometry, a 50 mm measurement aperture, illuminant D65, and a 10° standard observer. The instrument was calibrated with the manufacturer’s white calibration plate. Three readings were taken per MPP sample. The CIE Lab* coordinates were recorded; chroma (C*) and hue angle (h°) were calculated from a* and b*.

### 2.4. Preparation of Cream Cheese Formulations

**Figure 1:**
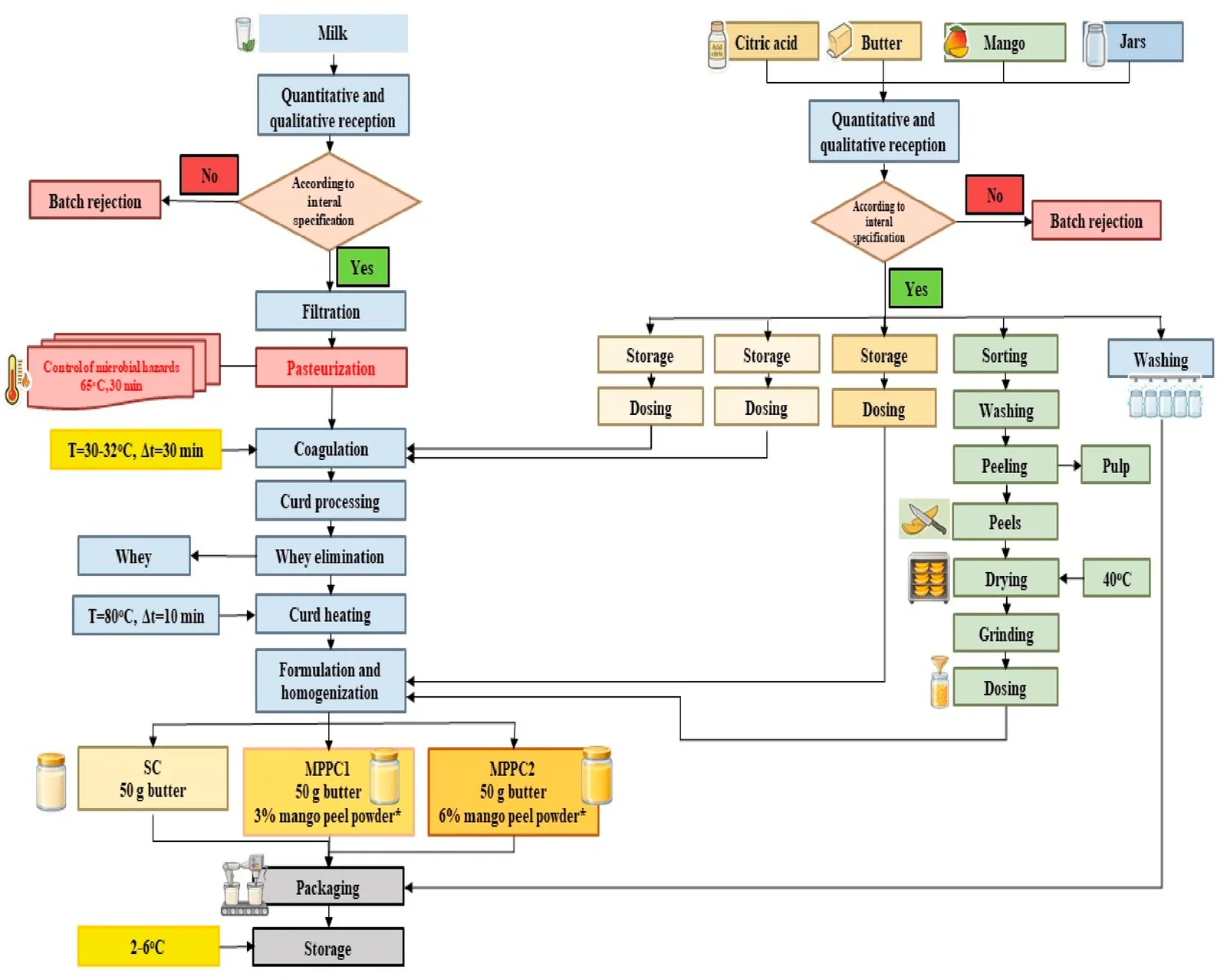
Process flow diagram for the preparation of control and mango peel powder-enriched spreadable cream cheese. Note: MPP, mango peel powder; SC, control cream cheese without MPP; MPPC1 and MPPC2, cream cheese prepared at nominal MPP addition levels of 3% and 6%, respectively; T, temperature; Δt, processing time; *w*/*w*, mass ratio. The nominal addition levels correspond to 3 and 6 g MPP per 100 g of the curd–butter mixture before MPP addition. Each portion of heated curd received 50 g of butter. Pasteurization at 65 °C for 30 min was applied to control microbial hazards associated with raw milk.

Three spreadable cream cheese formulations were prepared in three independent production batches: an unsupplemented control (SC) and two formulations supplemented with mango peel powder (MPP), designated MPPC1 and MPPC2. MPP was added at nominal levels of 3 and 6 g per 100 g of the curd–butter mixture before powder addition, respectively. These levels were selected based on previous studies and preliminary formulation considerations to evaluate the effect of MPP concentration on the physicochemical, functional, structural, and sensory properties of the final product. The preparation process is shown in Figure 1.

Milk was obtained from the university farm. According to the accompanying quality certificate, its fat and protein contents were 4.1% and 3.3%, respectively, its solids-not-fat content was 8.9%, and its titratable acidity was 15 °Th. Carbohydrate content was not reported separately on the certificate.

The milk was filtered and pasteurized in batches in an open vat at 65 °C for 30 min, then cooled to 30–32 °C. Citric acid was added at 3 g/L of milk, and the mixture was held for approximately 30 min to allow coagulation. The curd was mechanically processed to separate the whey and subsequently heated at 80 °C for 10 min. The heated curd was divided into portions for the three formulations, and 50 g of butter was added to each portion. MPP was then added to MPPC1 and MPPC2 at the specified rates relative to the mass of the corresponding curd–butter mixture. Each formulation was mixed separately for 2 min at a low-speed setting using a planetary mixer with a 40 L bowl capacity and a rated power of 1500 W. The cheeses were portioned into food-grade glass containers and refrigerated at 2–6 °C until analysis.

### 2.5. Physicochemical and Phytochemical Analysis of Cream Cheese

Moisture content was determined by oven drying, following the procedure described by Usturoi et al. [45]. Fat content was determined by Soxhlet extraction using a SER 148/3 system (Velp Scientifica, Usmate, Italy) [46,47], and protein content was determined by the Kjeldahl method using a UDK 149 analyser (Velp Scientifica, Milan, Italy) [48]. Crude fiber was determined by sequential acid and alkaline digestion using a Fibertec 2010 analyser (Tecator, Höganäs, Sweden) [49], and ash content was determined using a Caloris CL 1206 furnace (Caloris, Bucharest, Romania). [50]. Carbohydrate content and energy values were calculated according to [51,52]. The pH was measured using a WTW InoLab meter (Xylem Analytics GmbH, Weilheim, Germany) [48].

Total flavonoid content, total polyphenol content, and ABTS antioxidant activity were determined in SC, MPPC1, and MPPC2 using the procedures described in Section 2.3. Total carotenoids were analysed in all formulations; however, SC was below the LOQ and was therefore reported as <LOQ and excluded from quantitative comparison. For the storage experiment, samples were maintained in light-protected glass containers at 4 °C, and these phytochemical and antioxidant measurements were performed on days 0 and 21 [53].

### 2.6. Instrumental Colour and Texture

Cream cheese colour was measured according to Ciobotaru et al. [54] using the colorimeter and settings described in Section 2.3. Five readings were taken at different surface locations of each sample and averaged at the level of the independent production batch before statistical analysis. Chroma was calculated as C* = √(a^2^ + b^2^), and hue angle was calculated as h° = atan2(b*, a*) × 180/π [55]. Total colour difference was calculated as ΔE = √[(ΔL*)^2^ + (Δa*)^2^ + (Δb*)^2^] between each MPP-containing formulation and the control measured at the same storage time [56].

Texture profile analysis was performed at day 0 using a Mark-10 ESM300 motorized test stand (Mark-10 Corporation, Copiague, NY, USA) fitted with a cylindrical TA4 probe (38.1 mm diameter, 20 mm height), following a modified procedure described in [57]. Cylindrical cheese samples (approximately 30 mm in diameter and 40 mm in height) were subjected to two compression cycles to 50% of their initial height at a test speed of 1.0 mm/s, with a 0.05 N trigger force and a 5 s interval between cycles. Hardness was defined as the maximum force during the first compression (N), and cohesiveness as the ratio of the positive areas under the second and first compression curves (dimensionless). Springiness was calculated as the ratio of sample recovery during the second cycle to the corresponding displacement during the first cycle and was therefore dimensionless. Gumminess was calculated as hardness × cohesiveness (N), and chewiness as gumminess × springiness (N). Adhesiveness was determined from the negative area of the withdrawal curve and expressed in mJ.

### 2.7. Mineral and Microstructural Analyses

Mineral concentrations were determined using a ContrAA 700 atomic absorption spectrometer (Analytik Jena, Jena, Germany) [58]. Calcium, phosphorus, potassium, magnesium, zinc, and iron were reported for MPP as mg/100 g dw. Calcium, phosphorus, potassium, magnesium, sodium, and zinc were reported for cheese as mg/100 g sample.

MPP and cheese microstructures were examined by scanning electron microscopy (SEM) using a Quanta 450 microscope (FEI, Thermo Fisher Scientific, Hillsboro, OR, USA) equipped with an EDAX energy-dispersive X-ray spectroscopy (EDS) system (AMETEK Inc., Berwyn, PA, USA). MPP samples were mounted on aluminium stubs. Fresh cream cheese samples were spread directly onto aluminium stubs as approximately 2 mm-thick layers, without a separate drying step before loading into the microscope chamber. Samples were examined under low vacuum at 500× magnification and an accelerating voltage of 15 kV. Images included a 50 µm scale bar. EDS spectra were processed using TEAM software (version 4.1, EDAX Inc.) following calibration with an Al–Cu reference standard.

### 2.8. Sensory Evaluation

Thirty untrained participants evaluated appearance; colour and flavour intensity; acid, cream and mango odours; bitter, buttery, acid and sweet tastes; firmness; mouthfeel; aftertaste; and overall acceptability using seven-point scales [59]. Higher scores indicated greater intensity for intensity attributes; overall acceptability was assessed as an acceptance rating. Written informed consent was obtained from all participants. The study protocol was approved by the Ethics Committee of the Faculty of Agriculture, Iași University of Life Sciences (approval no. 973/06.07.2026).

### 2.9. Statistical and Multivariate Analysis

Results are presented as mean ± standard deviation (SD). Statistical analyses were performed using Minitab version 19.0 (Minitab, LLC, State College, PA, USA). Formulation effects for variables measured at a single time point were assessed by one-way analysis of variance (ANOVA). For variables measured on days 0 and 21, two-way ANOVA assessed the effects of formulation, storage time, and their interaction. Significant effects were followed by Tukey’s multiple-comparison test (*p* < 0.05). Principal component analysis (PCA) was performed using XLSTAT 2024 (Addinsoft, Paris, France).

## 3. Results and Discussion

### 3.1. Characterization of MPP

The composition of mango peel powder (MPP) was assessed to characterize the ingredient before its incorporation into spreadable cream cheese. Its phytochemical, antioxidant, proximate, and mineral characteristics are presented together in Table 1.

MPP contained 4.52 ± 0.29 mg GAE/g dw of total polyphenols, 1.15 ± 0.25 mg CE/g dw of total flavonoids, and 1.31 ± 0.21 mg/g dw of total carotenoids. Its ABTS antioxidant activity was 1074.24 ± 1.64 µmol TE/g dw, with 81.23 ± 0.32% radical inhibition. Previous studies have likewise identified phenolic compounds and other phytochemicals in mango peel, although their measured concentrations and antioxidant activity values vary with the drying and extraction procedures [11,60,61,62,63,64]. Direct comparison of antioxidant values across studies also requires attention to the assay and the basis used to express results [62].

Crude fiber accounted for 32.58 ± 0.24% of MPP, while carbohydrates accounted for 48.39 ± 0.35%. The proximate values were close to those reported by Jakha et al. for powder from the Langra cultivar [65]. Differences between cultivars and processing conditions remain relevant: Plaitho et al. reported a higher crude-fiber content for dried Fah-Lan mango peel powder [66]. Mango peel powders have also been described as sources of fiber and phytochemicals for food formulations [34].

Potassium was the predominant mineral measured in MPP (961.00 ± 0.36 mg/100 g dw), followed by calcium (259.50 ± 0.38 mg/100 g dw), phosphorus (145.50 ± 0.19 mg/100 g dw), and magnesium (118.25 ± 0.32 mg/100 g dw). Zinc and iron occurred at lower concentrations. Previous studies have reported mineral constituents in mango peel and variation in mango mineral composition among cultivars [15,67]. These differences should be considered when comparing powders from different raw materials. Reviews of mango by-product utilization also identify the peel as a potential source of recoverable phytochemical and mineral constituents [68].

The instrumental colour parameters of MPP are presented separately in Table 2 because they describe its appearance rather than its composition.

MPP had an L* value of 50.64 ± 0.13 and positive a* and b* values, indicating red and yellow colour components. Its hue angle of 70.29° placed the powder in the yellow-orange region of the CIELAB colour space.

### 3.2. Physicochemical Characterization of MPP-Enriched Cream Cheese

The cream cheese formulations differed in moisture content and in several compositional characteristics (Table 3). The most pronounced formulation-dependent changes were observed in moisture, crude fiber, and carbohydrates, whereas fat and protein showed smaller numerical increases. Calculated energy values also increased across the formulations.

According to the statistical groupings presented in Table 3, moisture, crude fiber, carbohydrates, and calculated energy values differed among all three formulations (*p* < 0.05). Fat content was higher in both supplemented formulations than in SC (*p* < 0.05), with no significant difference between MPPC1 and MPPC2 (*p* > 0.05). For protein, MPPC2 differed significantly from SC (*p* < 0.05), whereas MPPC1 did not differ significantly from either formulation (*p* > 0.05). Ash content and pH did not differ significantly among formulations (*p* > 0.05).

Moisture content decreased from 60.72 ± 0.07% in SC to 55.69 ± 0.11% in MPPC1 and 51.97 ± 0.06% in MPPC2. The corresponding total solids contents, calculated as 100 − moisture content, were 39.28%, 44.31%, and 48.03%, respectively. These differences should not be attributed solely to the dry matter introduced through MPP addition. Previous studies have described the influence of milk heat treatment, syneresis, and curd heating on cheese moisture and mass losses [69,70,71]. The lower measured moisture contents establish a compositional difference among formulations, but formulation-specific yields and water losses were not determined; therefore, a complete mass balance explaining the magnitude of this difference could not be established.

Crude fiber was reported as 0.00% in SC and increased to 1.54 ± 0.08% in MPPC1 and 3.05 ± 0.10% in MPPC2. The approximately two-fold difference between the supplemented formulations is consistent with the higher nominal MPP addition level and the powder’s crude-fiber content of 32.58%. Carbohydrates increased from 4.00 ± 0.06% in SC to 5.50 ± 0.02% in MPPC1 and 7.00 ± 0.08% in MPPC2. The higher fiber and carbohydrate contents are consistent with incorporation of plant-derived solids, although the lower moisture contents also affect concentrations expressed per unit mass of product. The possible influence of MPP on water distribution within the dairy matrix was not measured directly.

Fat content increased from 19.33 ± 0.09% in SC to 20.11 ± 0.07% in MPPC1 and 20.22 ± 0.04% in MPPC2. This modest increase on a product-mass basis coincided with lower moisture contents in the supplemented formulations. Calculated as [fat content/(100 − moisture content)] × 100, the corresponding fat proportions on a dry-matter basis were approximately 49.21%, 45.38%, and 42.10%, respectively. Thus, the higher fat percentages in the supplemented cheeses did not represent enrichment of the dry matter in fat. Each formulation received 50 g of butter, while MPP contained only 3.79% lipids. Fagan et al. [72] demonstrated that processing conditions can influence curd moisture, fat retention, and yield. However, the specific contributions of these factors to the observed differences could not be established in the present study because formulation-specific yields and component recoveries were not determined.

Protein content increased numerically from 15.11 ± 0.12% in SC to 16.24 ± 0.13% in MPPC1 and to 16.85 ± 0.18% in MPPC2. According to the groupings in Table 3, only the difference between SC and MPPC2 was significant (*p* < 0.05). Because MPP contained 6.32% protein, the higher protein percentages in the supplemented cheeses should not be attributed exclusively to direct protein enrichment by the powder. Differences in moisture and compositional balance must also be considered. Ebid et al. reported changes in moisture, protein, fat, and ash following MPP incorporation into Karish cheese [73], while mango peel powder has also been investigated in white soft cheese [74]. Other plant-derived ingredients have also produced formulation-dependent changes in processed cheese [75]. These studies provide context for formulation-dependent changes, although differences in cheese type and processing limit direct numerical comparisons.

The pH decreased numerically from 5.88 ± 0.04 in SC to 5.74 ± 0.05 in MPPC1 and 5.63 ± 0.06 in MPPC2, without significant differences among formulations according to Table 3 (*p* > 0.05). A numerical decrease in pH following MPP supplementation has also been reported in Karish cheese [73], and pH changes have been described in cream cheese containing *Citrus junos* peel powder [29]. Although pH can influence the protein network and mechanical properties of cream cheese [31], the present results do not establish that pH caused the texture differences among formulations.

Ash content ranged from 0.84 ± 0.05% to 0.92 ± 0.07%, with no significant differences among formulations (*p* > 0.05). Therefore, despite the mineral content of MPP, total ash content was not significantly different among the tested formulations. This finding should be distinguished from the results for individual mineral elements, which are discussed separately.

Calculated energy values increased from 250.41 kcal/100 g in SC to 267.95 kcal/100 g in MPPC1 and 277.38 kcal/100 g in MPPC2, equivalent to 1047.72, 1121.10, and 1160.56 kJ/100 g, respectively. These values were calculated using 9 kcal/g for fat and 4 kcal/g for protein and carbohydrates, without assigning an additional energy contribution to crude fiber. The higher values reflect the combined changes in the reported macronutrient concentrations and should not be attributed to MPP alone.

The supplemented formulations had lower moisture and higher crude-fiber and carbohydrate contents than SC. Fat content was higher in both supplemented formulations, whereas the protein difference was significant only between SC and MPPC2 according to the current statistical groupings. Ash and pH remained statistically unchanged. These compositional results provide context for the colour and texture differences discussed below, while the absence of a complete formulation mass balance limits attribution of each change to a specific mechanism.

### 3.3. Phytochemical Profile and Antioxidant Activity During Refrigerated Storage

MPP incorporation was associated with higher flavonoid and polyphenol contents and greater ABTS antioxidant activity than those measured in the control formulation (Table 4). Among the two formulations analysed for carotenoids, MPPC2 showed higher measured concentrations than MPPC1 at both storage times. Phytochemical concentrations decreased numerically during 21 days of refrigerated storage. ABTS antioxidant activity decreased only slightly during storage, although the day effect was statistically significant according to the current groupings.

The SC sample was analysed using the same method, but its absorbance response was below the LOQ; therefore, SC was reported as <LOQ and excluded from quantitative carotenoid comparisons. Total carotenoids were quantified only in MPPC1 and MPPC2. At day 0, their concentrations were 0.38 ± 0.23 and 0.57 ± 0.26 mg/g dw, respectively. After 21 days, the corresponding values were 0.31 ± 0.20 and 0.49 ± 0.25 mg/g dw, giving numerical retentions of 81.58% and 85.96%.

Total flavonoids increased across the formulations from 0.27 ± 0.15 mg CE/g dw in SC to 0.46 ± 0.16 mg CE/g dw in MPPC1 and to 0.55 ± 0.18 mg CE/g dw in MPPC2 at day 0. After storage, the respective values were 0.20 ± 0.09, 0.38 ± 0.11, and 0.47 ± 0.15 mg CE/g dw. The calculated retentions were 74.07%, 82.61%, and 85.45%.

Total polyphenols followed the same formulation ranking. At day 0, concentrations were 0.85 ± 0.11 mg GAE/g dw in SC, 1.15 ± 0.15 mg GAE/g dw in MPPC1, and 1.66 ± 0.18 mg GAE/g dw in MPPC2. They decreased to 0.72 ± 0.10, 1.01 ± 0.13, and 1.51 ± 0.14 mg GAE/g dw after 21 days. MPPC2 retained the highest measured concentration and had the highest numerical polyphenol retention (90.96%) among the three formulations.

ABTS antioxidant activity was also highest in MPPC2 throughout storage. At day 0, values were 505.20 ± 12.42, 590.10 ± 15.25, and 672.21 ± 18.21 µmol TE/g dw for SC, MPPC1, and MPPC2, respectively. After 21 days, they were 502.68 ± 11.36, 587.88 ± 13.21, and 669.78 ± 14.58 µmol TE/g dw, corresponding to calculated retentions of 99.50–99.64%.

The higher flavonoid and polyphenol contents and ABTS activity of the supplemented cheeses are consistent with studies identifying mango peel as a source of phytochemicals [75,76,77,78]. Ebid et al. also reported increased phenolic and flavonoid contents and antioxidant activity in peel-supplemented Karish cheese [73]; mango peel powder has likewise been investigated in white soft cheese [74]. The numerical decreases in phytochemical concentrations during storage may be influenced by interactions between plant-derived phenolics and milk proteins, as observed in another refrigerated dairy matrix [79]. However, the mechanisms responsible for the changes in the present formulations were not investigated directly. Overall, MPPC2 maintained the highest measured carotenoid concentration among the two formulations analysed for that parameter, as well as the highest flavonoid and polyphenol contents and ABTS activity among the three formulations.

### 3.4. Instrumental Colour Characteristics During Refrigerated Storage

The incorporation of MPP produced a clear concentration-dependent modification of the colour profile of the cream cheese formulations (Table 5). The most pronounced effect was observed immediately after formulation, with increasing MPP concentration reducing lightness and shifting the chromatic coordinates toward a more saturated yellow-orange appearance. Further, comparatively smaller changes occurred during the 21-day refrigerated-storage period.

The yellow-orange colour of MPP is consistent with the changes observed in the supplemented cheeses. Similar effects of mango peel ingredients on product colour have been reported in other foods [24,25,80], while plant powders have also modified instrumental colour parameters in dairy products [28,81].

At day 0, the control formulation showed the highest lightness (L* = 90.04 ± 0.21). MPP incorporation reduced L* to 81.83 ± 0.20 in MPPC1 and 76.20 ± 0.16 in MPPC2, showing that the darkening of the cream cheese matrix increased with the amount of powder incorporated. After 21 days, L* decreased further to 88.68 ± 0.18, 79.59 ± 0.17, and 74.54 ± 0.15 in SC, MPPC1, and MPPC2, respectively. The effect of the formulation was therefore considerably larger than the additional change occurring during refrigerated storage.

The a* coordinate provided further evidence of the chromatic shift induced by MPP. The control retained slightly negative a* values (−2.19 at day 0 and −1.98 at day 21), whereas both supplemented formulations displayed positive values. At day 0, a* reached 2.62 in MPPC1 and 4.53 in MPPC2 and increased to 3.02 and 5.11, respectively, after storage. Thus, addition of MPP shifted the dairy matrix away from the slightly green component of the control and toward the red component of the CIELAB colour space.

An analogous response was observed for b*, which increased from 19.44 in SC to 23.04 in MPPC1 and 25.95 in MPPC2 at day 0. After 21 days, the corresponding values were 20.32, 24.31, and 27.21. This progressive increase in yellowness is consistent with the chromatic characteristics of MPP and with the presence of naturally coloured constituents, including carotenoid-type pigments, in mango peel. The contribution of plant pigments to colour changes in cream cheese has also been reported for *Citrus junos* peel powder, where supplementation reduced lightness and increased the red and yellow components of the product [29]. Similar changes in objective colour parameters have been reported for dairy matrices containing fruit-derived ingredients [82,83].

Chroma followed the same general pattern. At day 0, C* increased from 19.56 in the control to 23.19 and 26.34 in MPPC1 and MPPC2, respectively. Values after refrigerated storage were 20.42, 24.50, and 27.69. The higher chroma values of the MPP-containing formulations indicate greater colour saturation, with MPPC2 showing the most intense colour at both sampling times.

The hue angle provided values of 96.43° and 95.57° for SC, 83.51° and 82.92° for MPPC1, and 80.10° and 79.36° for MPPC2 at days 0 and 21, respectively. The control was therefore located slightly beyond the nominal yellow region toward green-yellow, whereas MPP incorporation progressively shifted the hue below 90°, toward a warmer yellow-orange appearance. Within each formulation, the change in hue angle between day 0 and day 21 was small, suggesting that storage altered colour intensity more markedly than the fundamental hue of the samples.

The overall magnitude of the colour modification was further demonstrated by ΔE. Relative to the control measured at the same storage time, MPPC1 showed ΔE values of 10.17 at day 0 and 11.12 at day 21, whereas values for MPPC2 were 16.71 and 17.26, respectively. Thus, even the 3% supplementation level produced a marked instrumental differentiation from the control, while incorporation of 6% MPP resulted in a considerably larger colour deviation. The slight increase in ΔE after storage further indicates that the visual separation between the enriched formulations and the control became somewhat greater over time.

Marcillo-Parra et al. [84] identified carotenoids and several phenolic compounds in mango peel and demonstrated substantial variation in these compounds among mango cultivars [84]. Such constituents, together with the intrinsic colour of the dried peel particles, provide a plausible explanation for the lower L* and higher a*, b*, and C* values observed after MPP incorporation.

The colour data show that MPP concentration was the main determinant of the visual differentiation among the cream cheese formulations. Increasing MPP from 3% to 6% resulted in lower lightness, greater redness and yellowness, higher saturation, and a progressively warmer hue. Refrigerated storage produced additional but smaller changes in these parameters.

### 3.5. Instrumental Texture Profile

The incorporation of MPP markedly changed the instrumental texture profile of the cream cheese formulations (Table 6). All measured parameters increased numerically with MPP concentration, with the largest differences from the control generally observed for the formulation containing 6% MPP. Instrumental texture was compared among formulations at day 0 (Table 6).

Hardness increased from 4.38 ± 0.18 N in SC to 5.59 ± 0.21 N in MPPC1 and to 7.09 ± 0.26 N in MPPC2. Relative to the control, these values correspond to numerical increases of approximately 27.6% and 61.9%, respectively. The response indicates that progressively higher MPP incorporation produced a firmer cream cheese matrix.

Adhesiveness showed a similar concentration-dependent increase, from 0.46 ± 0.15 mJ in SC to 0.70 ± 0.17 mJ in MPPC1 and 0.94 ± 0.18 mJ in MPPC2. Thus, the work associated with separation of the sample from the probe approximately doubled between the control and the 6% MPP formulation. In a spreadable cheese system, this change is relevant because an increase in adhesiveness may affect both instrumental handling properties and the perception of stickiness during consumption.

The simultaneous increases in hardness and adhesiveness are not necessarily contradictory, because hardness describes resistance to compression, whereas adhesiveness describes the work required for probe detachment. A comparable increase in both parameters has been reported in low-fat cream cheese supplemented with plant-derived gums [85]. In the present formulations, increasing MPP addition also coincided with lower measured moisture contents. These compositional and textural changes occurred together, although the individual effects of moisture and MPP addition were not isolated.

Cohesiveness increased from 0.39 ± 0.12 in SC to 0.50 ± 0.13 in MPPC1 and 0.61 ± 0.17 in MPPC2. Springiness followed the same numerical pattern, increasing from 0.41 ± 0.10 to 0.52 ± 0.15 and 0.69 ± 0.20, respectively. These changes suggest that the supplemented matrices exhibited a greater capacity to maintain their internal structure and partially recover after deformation.

The texture response may be associated with the incorporation of a particulate, fiber-rich plant ingredient into the protein–fat matrix. MPP contained 32.58% crude fiber, and increasing its concentration in the formulation raised the measured fiber content of the finished cheese. Fiber and other insoluble constituents can interact physically with the continuous dairy phase and influence water distribution and matrix packing.

Similar formulation-dependent changes have been reported for dairy matrices containing plant-derived ingredients. Previous work with whey cheese supplemented with plant by-product powders showed that increasing the amount of added material altered the texture profile, while increased hardness and springiness have also been described in Ricotta cheese containing *Spirulina platensis* [43,86]. These observations support the broader view that incorporation of solids can alter the mechanical behaviour of soft cheese matrices, although the direction and magnitude of the response depend on the composition and physical properties of the added ingredient.

Gumminess increased more strongly than hardness alone, from 1.71 ± 0.23 N in SC to 2.80 ± 0.26 N in MPPC1 and 4.32 ± 0.28 N in MPPC2. These values are internally consistent with the relationship between hardness and cohesiveness used in texture profile analysis. Similarly, chewiness increased from 0.70 ± 0.17 N in the control to 1.45 ± 0.19 N and 2.98 ± 0.29 N in MPPC1 and MPPC2, respectively, and was consistent with the combined changes in gumminess and springiness.

Shelke et al. [87] reported decreases in several texture parameters in low-fat soft cheese supplemented with spray-dried jamun juice powder, where the formulation also contained carrier materials that promoted water retention and a softer matrix [87]. Other fruit-derived ingredients have likewise been associated with lower cream cheese hardness when their incorporation increased moisture or disrupted the protein network [88]. These contrasting results emphasize that texture is governed by the combined effects of ingredient composition, particle characteristics, hydration behaviour, and interactions with the dairy matrix rather than by the presence of plant material alone [89,90].

The progressive increase in hardness, adhesiveness, gumminess, and chewiness indicates that 6% MPP produced the strongest modification of the mechanical properties of the cream cheese. The 3% formulation showed an intermediate texture profile, suggesting a less pronounced departure from the control. This distinction becomes particularly relevant when the instrumental results are considered alongside microstructure and sensory evaluation: The higher firmness of MPPC2 coincided with a more heterogeneous matrix and a stronger sensory perception of firmness, whereas MPPC1 retained the highest overall sensory acceptability.

### 3.6. Mineral Composition

The mineral composition of the cream cheese formulations was affected by the incorporation of MPP (Table 7). The clearest formulation-dependent changes were observed for phosphorus, potassium, and magnesium, whereas calcium and zinc increased more moderately. In contrast, sodium decreased progressively as the MPP level increased.

Calcium content increased progressively from 97.02 ± 0.34 mg/100 g in the control to 100.87 ± 0.36 mg/100 g in MPPC1 and 104.75 ± 0.37 mg/100 g in MPPC2. These changes correspond to increases of approximately 4.0% and 8.0%, respectively, relative to SC. The moderate response is compatible with both the mineral contribution of MPP and the already appreciable calcium content of the dairy matrix. Calcium remains closely associated with the protein structure of cheese, and its concentration can be influenced by both formulation and processing conditions [91,92].

Phosphorus showed a stronger relative increase, rising from 24.07 ± 0.24 mg/100 g in SC to 28.13 ± 0.25 mg/100 g in MPPC1 and 32.29 ± 0.27 mg/100 g in MPPC2. These values represent increases of approximately 16.9% and 34.2% compared with the control. This trend is compatible with the phosphorus content measured in MPP and indicates that the plant ingredient contributed measurably to the mineral profile of the supplemented formulations.

Potassium was the most abundant of the minerals that increased following supplementation. Its concentration rose from 132.11 ± 0.30 mg/100 g in SC to 143.79 ± 0.33 mg/100 g in MPPC1 and to 155.58 ± 0.36 mg/100 g in MPPC2, corresponding to increases of approximately 8.8% and 17.8%. This result is consistent with the mineral composition of MPP, where potassium was the predominant mineral at 961 mg/100 g. Mango peel has also been described as a potassium-rich by-product, and increased potassium concentrations have been reported when mango peel were incorporated into food matrices [76,80].

The largest relative increase among the measured minerals was observed for magnesium. Its concentration increased from 9.56 ± 0.20 mg/100 g in SC to 13.70 ± 0.26 mg/100 g in MPPC1 and 18.39 ± 0.29 mg/100 g in MPPC2. Relative to the control, these values correspond to increases of approximately 43.3% and 92.4%, respectively.

Zinc increased from 0.50 ± 0.06 mg/100 g in SC to 0.54 ± 0.08 mg/100 g in MPPC1 and 0.57 ± 0.10 mg/100 g in MPPC2. In contrast to the other minerals, sodium decreased as MPP concentration increased. Values declined from 314.00 ± 0.17 mg/100 g in SC to 304.64 ± 0.19 mg/100 g in MPPC1 and 295.28 ± 0.25 mg/100 g in MPPC2, representing decreases of approximately 3.0% and 6.0%, respectively. The magnitude of this decrease closely follows the level of MPP incorporation and may reflect partial dilution of the sodium-containing dairy fraction by the plant ingredient. The overall pattern is consistent with previous studies showing that the incorporation of mineral-containing plant ingredients can modify the mineral composition of cheese. Pawlos et al. emphasized the sensitivity of mineral composition in fresh cheese to formulation and processing conditions [92], while Ali et al. reported increased concentrations of Ca, Fe, Mg, P, K, and Zn following the incorporation of plant-derived ingredients into Karish cheese [93]. The magnitude of such changes depends on both the mineral composition of the added ingredient and its concentration in the final formulation.

Incorporation of MPP shifted the mineral profile of the cream cheese toward higher concentrations of calcium, phosphorus, potassium, magnesium, and, to a lesser extent, zinc, while sodium decreased with increasing supplementation. The strongest relative response was observed for magnesium, whereas potassium remained quantitatively the most abundant mineral among those enhanced by MPP. These findings support a contribution of the plant ingredient to the mineral composition of the enriched cheese, although they should not be interpreted as evidence of improved mineral bioavailability, which was not investigated.

### 3.7. Microstructural and Elemental Surface Characterization

**Figure 2:**
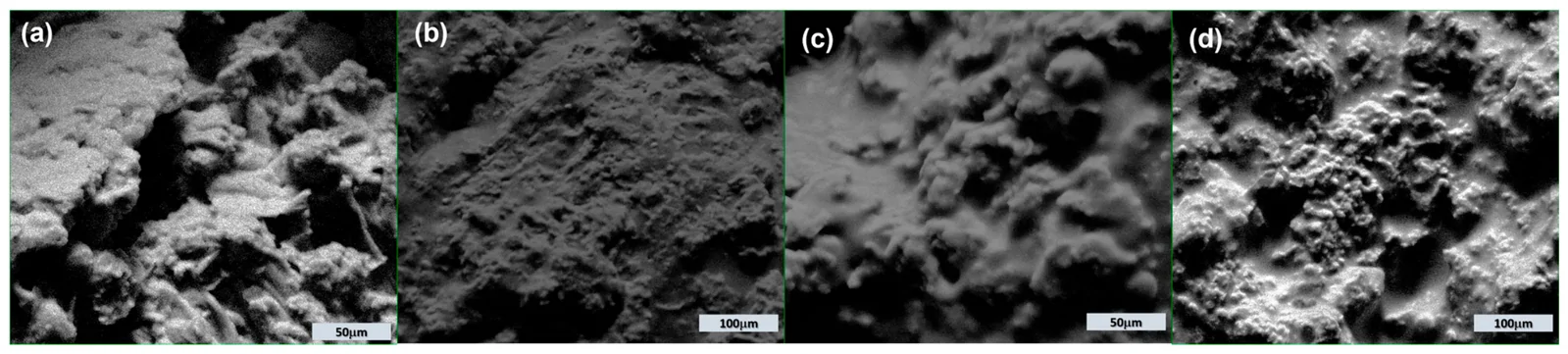
Scanning electron microscopy (SEM) micrographs of MPP (**a**), control cream cheese (SC) (**b**), cream cheese containing 3% MPP (MPPC1) (**c**), and cream cheese containing 6% MPP (MPPC2) (**d**).

Scanning electron microscopy was used to examine the morphology of MPP and to assess changes in the organization of the cream cheese matrix following MPP incorporation. Representative SEM micrographs of MPP, the control cream cheese, and the formulations containing 3% and 6% MPP are shown in Figure 2.

MPP displayed an irregular and heterogeneous morphology, with fragmented particles of variable dimensions and relatively rough surfaces (Figure 2a). This structure is consistent with mechanically ground plant material containing disrupted cell-wall and fibrous components. The heterogeneous particle morphology is relevant to its incorporation into a semi-solid dairy matrix, since such particles can act as dispersed structural elements rather than becoming fully incorporated into the continuous protein–fat phase.

The control cream cheese exhibited a comparatively more continuous and compact microstructure (Figure 2b), with fewer distinguishable particulate regions. Fresh and spreadable cheeses are generally characterized by a complex matrix in which protein aggregates, fat domains, and aqueous regions coexist, and their spatial organization contributes to the mechanical properties of the product [94]. The morphology observed for SC is therefore consistent with the comparatively softer instrumental texture.

The incorporation of 3% MPP altered the surface organization of the cheese matrix (Figure 2c). Particulate regions became more evident and the structure appeared less homogeneous than that of SC, indicating the presence of dispersed plant material within the dairy phase. At this supplementation level, however, the matrix retained a relatively integrated appearance, with MPP particles distributed throughout the surrounding cheese structure.

A more pronounced modification was observed when the MPP concentration was increased to 6% (Figure 2d). MPPC2 exhibited greater surface roughness and structural heterogeneity, together with more evident particulate aggregates and discontinuous regions. The comparison between Figure 2c,d therefore suggests that the extent of microstructural modification increased with MPP concentration.

The higher fiber content of the supplemented formulations provides a plausible explanation for at least part of this structural response. Fibrous and other insoluble components introduced with MPP can influence particle–matrix interactions and the spatial organization of proteins and fat. At higher inclusion levels, closer contact among plant particles may also favour aggregation and reduce the apparent homogeneity of the dairy phase. Similar structural modifications have been reported when fiber-rich or plant-derived ingredients were incorporated into dairy and spreadable food matrices [2,95].

The progressive changes visible in Figure 2 are consistent with the instrumental texture results. Hardness increased from 4.38 N in SC to 5.59 N in MPPC1 and 7.09 N in MPPC2, while gumminess and chewiness followed the same concentration-dependent pattern. In particular, the more heterogeneous and particulate appearance of MPPC2 coincided with the strongest instrumental modification of texture. This agreement does not establish a direct causal relationship, but it supports the view that MPP incorporation altered both the physical organization and the mechanical behaviour of the cream cheese matrix.

**Figure 3:**
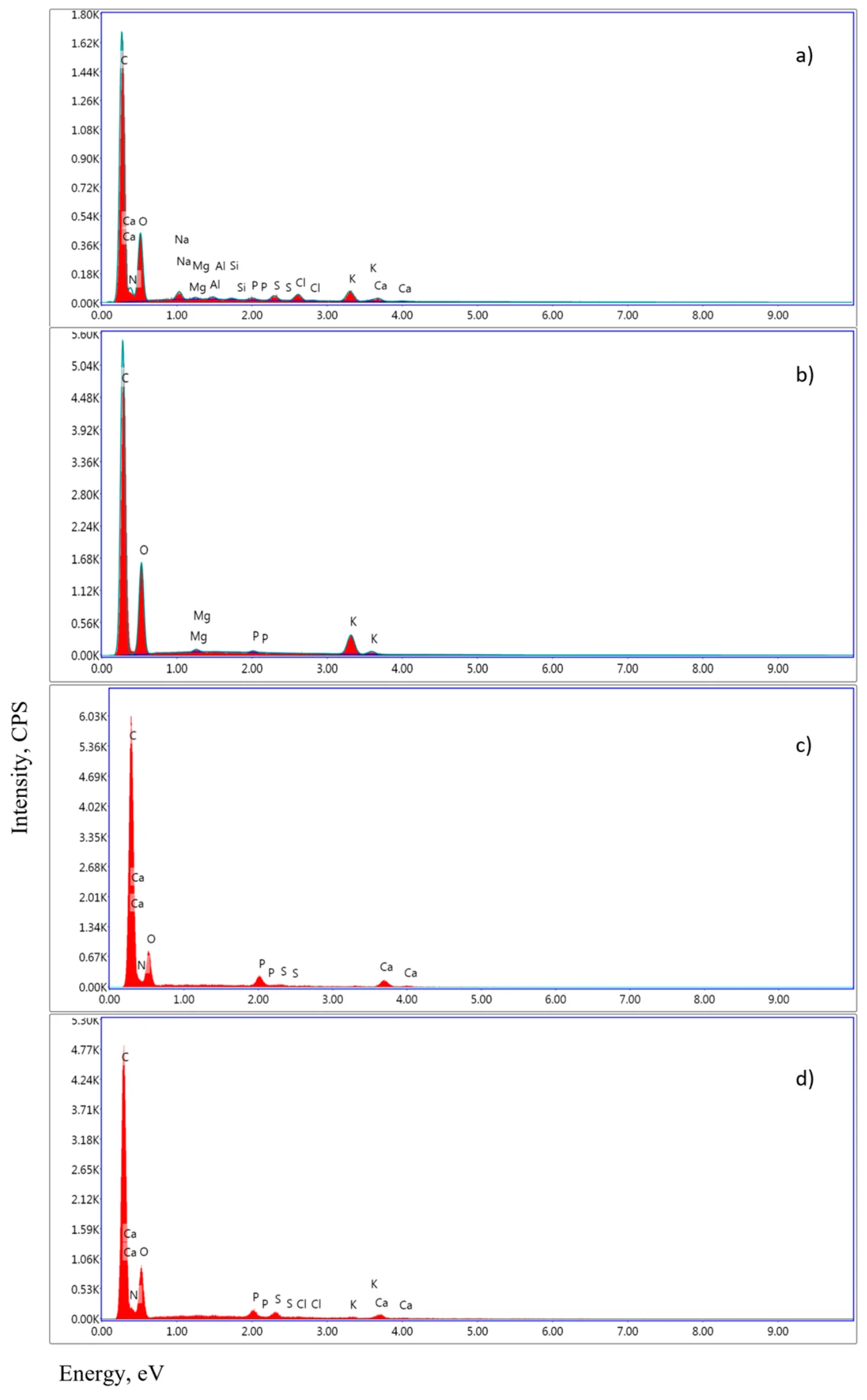
Energy-dispersive X-ray spectroscopy (EDS) spectra of MPP (**a**), control cream cheese (SC) (**b**), cream cheese containing 3% MPP (MPPC1) (**c**), and cream cheese containing 6% MPP (MPPC2) (**d**).

Energy-dispersive X-ray spectroscopy was used as a complementary surface analysis to identify the elemental signals associated with MPP and the cream cheese formulations. The corresponding EDS spectra are presented in Figure 3.

The spectra showed carbon and oxygen as the predominant signals in both MPP and the cream cheese samples (Figure 3a–d), as expected for materials composed primarily of organic constituents. Additional signals associated with mineral elements were detected in the cheese matrices. In particular, calcium and phosphorus signals are compatible with the mineral constituents of the dairy phase, while sulfur may originate from sulfur-containing components of milk proteins.

Comparison of the spectra in Figure 3b–d indicates that MPP incorporation was accompanied by changes in the relative elemental surface profile of the cream cheese samples.

The MPP spectrum shown in Figure 3a also confirms the presence of an organic plant-derived matrix together with detectable inorganic constituents. Differences between the MPP spectrum and those of the dairy formulations are therefore compatible with the incorporation of chemically and structurally distinct plant particles into the cheese matrix. Nevertheless, the EDS spectra alone cannot demonstrate mineral enrichment or establish the nutritional contribution of individual elements.

The SEM observations demonstrate a progressive modification of cream cheese microstructure with increasing MPP concentration. The 3% formulation retained a comparatively integrated matrix despite the presence of dispersed plant particles, whereas the 6% formulation showed greater heterogeneity, roughness, and particle aggregation. These structural observations are consistent with the concentration-dependent increase in instrumental firmness and with the stronger sensory perception of firmness subsequently observed for MPPC2. The microstructural and texture results indicate that moderate MPP incorporation produces a less pronounced disruption of the cream cheese matrix than the higher supplementation level.

### 3.8. Sensory Profile and Multivariate Analysis

**Figure 4:**
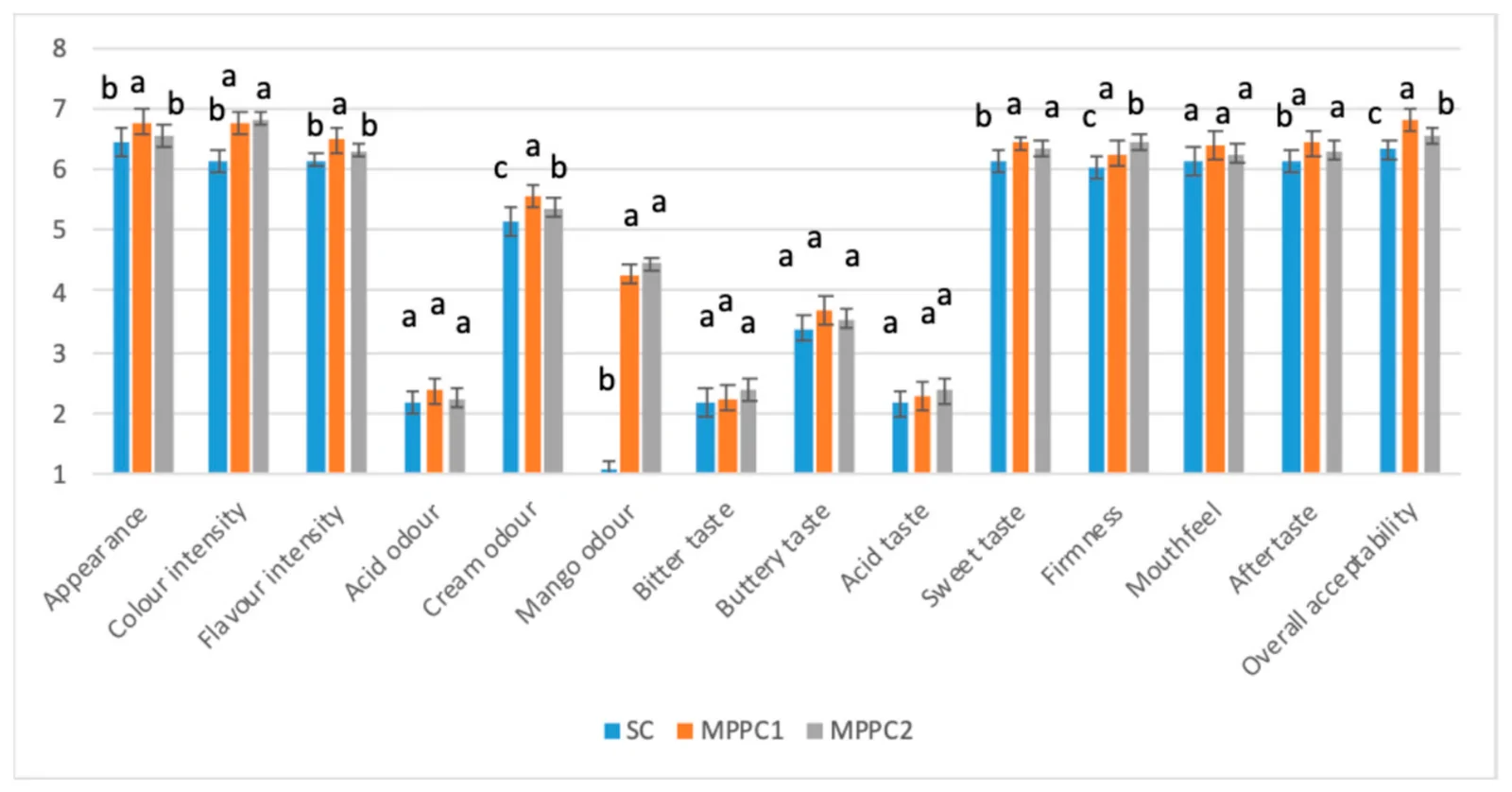
Sensory scores of the control and MPP-enriched cream cheese formulations evaluated on a seven-point scale. SC, control cream cheese; MPPC1 and MPPC2, cream cheese prepared with nominal MPP addition levels of 3 and 6 g/100 g of the curd–butter mixture before powder addition, respectively. Bars represent mean values, and error bars represent standard deviations. Within each sensory attribute, formulations sharing a lowercase letter do not differ significantly, whereas those with different letters differ significantly (*p* < 0.05).

A preliminary sensory evaluation was conducted to assess the effect of mango peel powder (MPP) incorporation on the perceived characteristics of the cream cheese formulations. Fourteen attributes covering appearance, odour, taste, texture, and overall acceptability were rated on a seven-point scale. Figure 4 presents the mean scores, their variability, and the statistical comparisons among formulations for each attribute.

The formulations showed different sensory profiles (Figure 4). MPPC1 had the highest numerical scores for appearance (6.76), flavour intensity (6.48), cream odour (5.55), buttery taste (3.67), sweet taste (6.43), mouthfeel (6.38), aftertaste (6.42), and overall acceptability (6.81). Overall acceptability was significantly higher for MPPC1 than for MPPC2 (6.52) and SC (6.32) (*p* < 0.05), whereas MPPC2 and SC did not differ significantly (*p* > 0.05). The highest numerical score did not indicate a significant difference for every attribute; buttery taste and mouthfeel, for example, did not differ significantly among formulations (*p* > 0.05).

MPPC2 received the highest scores for colour intensity (6.81), mango odour (4.45), and firmness (6.44). Its greater colour intensity and perceived firmness were consistent with the instrumental colour and texture measurements: MPPC2 had the greatest colour difference from SC and the highest instrumental hardness. The control contained no MPP and received a score close to the minimum of the scale for mango odour; this score should not be interpreted as evidence of a distinct mango odour in SC.

The relationship between sensory and instrumental colour measurements was also evident across the formulations. Increasing MPP concentration reduced instrumental lightness (L*) and increased the red–green (a*) and yellow–blue (b*) coordinates and chroma (C*), as discussed in Section 3.4. MPPC2 also received the highest sensory colour-intensity score. Similar changes in instrumental and perceived colour have been described for fresh cheeses containing plant-derived ingredients [96,97].

MPPC2 had the highest numerical scores for bitter taste (2.37) and acid taste (2.36). However, these attributes did not differ significantly among formulations (*p* > 0.05; Figure 4). MPPC1 received the highest numerical mouthfeel score, without significant differences among formulations, and significantly higher overall acceptability than SC and MPPC2 (*p* < 0.05). These results distinguish numerical differences in attribute intensity from the statistically significant difference in overall acceptability.

Sensory firmness increased alongside instrumental hardness, which was 4.38 N in SC, 5.59 N in MPPC1, and 7.09 N in MPPC2. The micrographs also showed greater structural heterogeneity at higher MPP concentrations (Figure 2d). However, MPPC1 received the highest mouthfeel score despite being less firm than MPPC2. Thus, the formulation with the greatest firmness did not receive the highest numerical mouthfeel rating. This distinction is relevant to spreadable cheeses, for which firmness and the overall oral perception of texture represent different characteristics [98].

**Figure 5:**
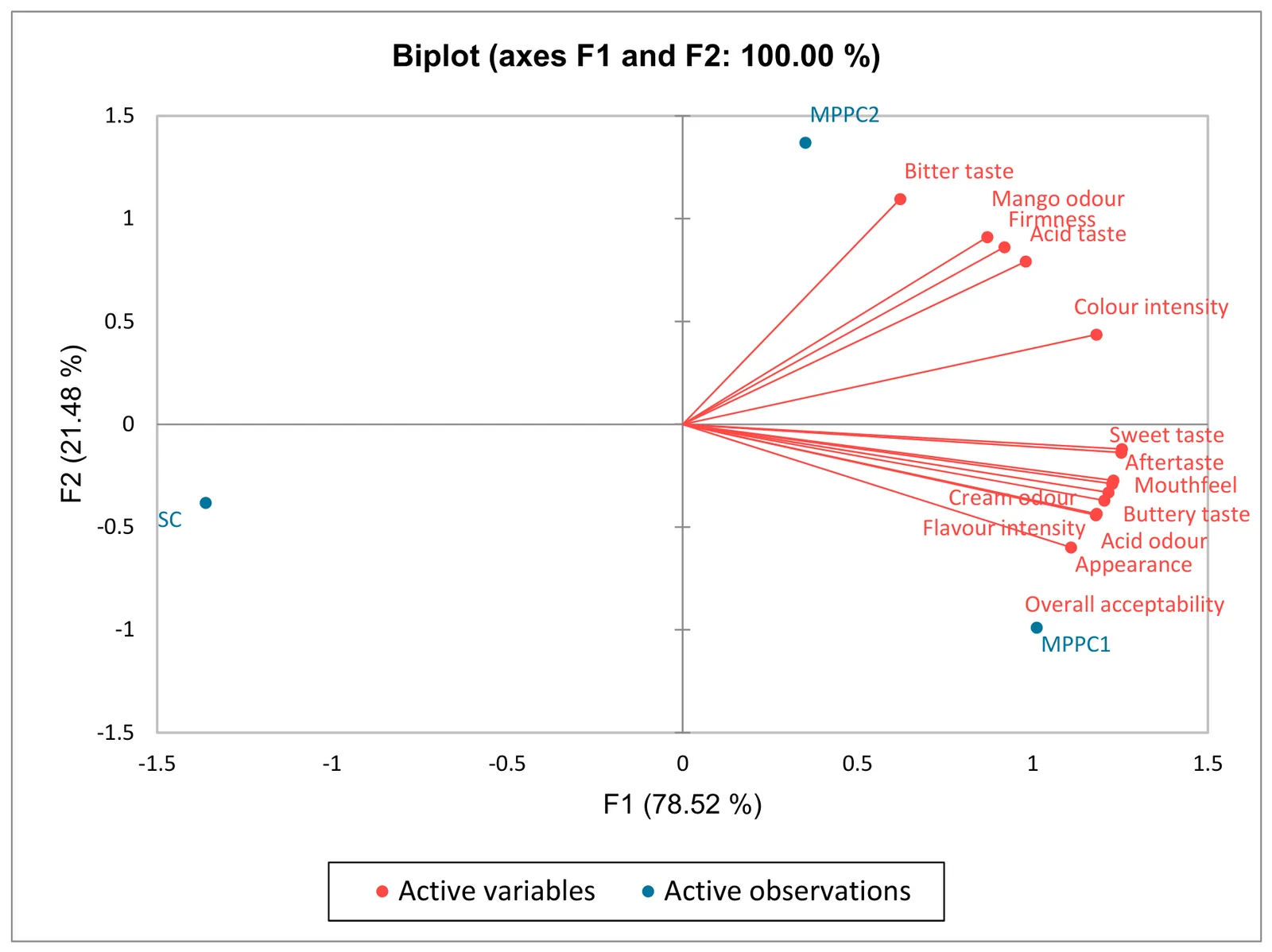
Principal component analysis (PCA) biplot based on the mean sensory scores of SC, MPPC1, and MPPC2. SC, control cream cheese; MPPC1 and MPPC2, cream cheese prepared with nominal MPP addition levels of 3 and 6 g per 100 g of the curd–butter mixture before powder addition, respectively.

Principal component analysis (PCA) was applied to formulation-level mean sensory scores to provide an exploratory visualization of the relationships among attributes and formulations (Figure 5). Because the analysis included only three formulation-level profiles, the resulting positions and associations were interpreted descriptively. PCA was not used to establish statistical significance or sensory acceptability.

The first principal component (F1) accounted for 78.52% of the variation in the three formulation-level profiles, and the second (F2) accounted for 21.48% (Figure 5). F1 separated SC from the MPP-containing formulations, while F2 differentiated MPPC1 and MPPC2. Because the analysis included only three formulation-level profiles, the PCA should be interpreted as a descriptive visualization of these samples.

In the biplot, MPPC1 was positioned closer to appearance, overall acceptability, flavour intensity, mouthfeel, buttery taste, cream odour, aftertaste, and sweet taste. MPPC2 was positioned closer to colour intensity, mango odour, firmness, bitter taste, and acid taste. These positions broadly reflect the numerical scores in Figure 4. Proximity in the biplot describes the pattern of the included mean scores; it does not establish which attributes caused the overall acceptability ratings.

The sensory scores indicate greater perceived colour intensity, mango odour, and firmness in MPPC2. MPPC1 obtained significantly higher overall acceptability than SC and MPPC2 (*p* < 0.05), while mouthfeel did not differ significantly among formulations despite its highest numerical score in MPPC1. PCA provided a complementary descriptive summary of the three sensory profiles.

### 3.9. Integrated Discussion

MPP incorporation was associated with changes in both the composition and quality characteristics of spreadable cream cheese. The supplemented formulations contained more crude fiber, flavonoids, and polyphenols and showed greater ABTS antioxidant activity than SC. MPPC2 had the highest measured carotenoid concentration among the two formulations analysed for this parameter. These compositional differences were accompanied by changes in appearance and texture, showing that the effects of MPP extended beyond the addition of phytochemicals. This combined compositional and technological perspective is consistent with reviews of by-product valorization in dairy foods [99].

The supplemented cheeses had lower measured moisture contents and higher instrumental hardness and adhesiveness. Gumminess and chewiness also increased, partly reflecting their mathematical dependence on hardness, cohesiveness, and springiness. Together, these observations describe a matrix that resisted compression more strongly and required greater work for probe detachment. The SEM images showed greater structural heterogeneity with increasing MPP addition. However, neither the micrographs nor the compositional measurements established the individual contributions of moisture, plant particles, and dairy components to the mechanical response.

Instrumental colour and sensory observations provided complementary information. The shift towards a darker, more yellow-orange appearance was accompanied by higher perceived colour intensity in MPPC2. Its greater instrumental hardness also coincided with the highest numerical sensory firmness score. These agreements support the interpretation that some instrumental differences were perceptible to the panel, although sensory ratings and instrumental measurements describe distinct properties.

The formulation with the strongest instrumental and phytochemical changes did not receive the highest numerical acceptance rating. MPPC1 obtained the highest numerical scores for overall acceptability, mouthfeel, and aftertaste, whereas MPPC2 showed greater colour intensity, firmness, and stronger bitter and acidic notes. This pattern suggests that an intermediate MPP addition level may provide a more favourable sensory balance. Nevertheless, because the sensory evaluation was preliminary and involved a limited panel, these findings should not be interpreted as establishing a broadly generalizable consumer preference or a definitive optimal supplementation level.

The storage results should be interpreted within their analytical scope. Phytochemical concentrations decreased numerically over 21 days, while ABTS antioxidant activity retained approximately 99.5–99.6% of its initial value. Colour was also evaluated during storage, whereas texture and sensory characteristics were assessed only at day 0. Consequently, the study demonstrates persistence of the measured antioxidant response but does not establish stability of the complete quality profile or microbiological safety throughout storage. Further work should combine verified formulation mass balances, microbiological assessment, repeated texture and sensory measurements, and matched batch-level data to evaluate the relationships among composition, structure, and acceptance.

## 4. Conclusions

Mango peel powder (MPP) showed potential as an ingredient for the compositional and phytochemical enrichment of spreadable cream cheese. At nominal addition levels of 3 and 6 g per 100 g of the curd–butter mixture before powder addition, the supplemented formulations had lower moisture and higher crude-fiber and carbohydrate contents than the control. Supplementation also increased measured flavonoid and polyphenol contents and ABTS antioxidant activity. Increasing MPP addition produced darker, more saturated yellow-orange cheeses with greater instrumental hardness, adhesiveness, gumminess, and chewiness. SEM images showed greater surface heterogeneity and particle aggregation in MPPC2, providing qualitative evidence of formulation-dependent structural differences.

During 21 days of refrigerated storage, the supplemented formulations maintained higher flavonoid and polyphenol contents and ABTS activity than the control, despite numerical decreases in phytochemical concentrations. Carotenoids were below the LOQ in SC and quantifiable only in MPPC1 and MPPC2; MPPC2 maintained the higher concentration among the enriched formulations. ABTS activity retention ranged from 99.50% to 99.64% across the three formulations. These storage findings concern phytochemical measurements, antioxidant activity, and colour; texture, microstructure, and sensory properties were assessed at day 0.

In the preliminary sensory evaluation, MPPC1 achieved the highest overall acceptability score (6.81/7), significantly exceeding SC and MPPC2 (*p* < 0.05). MPPC2 showed greater colour intensity, mango odour, and perceived firmness. The exploratory PCA provided a descriptive visualization of the sensory differences among formulations. Among the tested addition levels, MPPC1 combined compositional enrichment with the highest sensory acceptability, whereas MPPC2 provided greater phytochemical enrichment and more pronounced changes in appearance and texture.

Further research should establish formulation-specific yields and component recoveries to clarify the compositional changes, evaluate microbiological shelf life and the effectiveness of MPP treatment, and assess potential allergenic constituents and gastrointestinal bioaccessibility. Processing optimization and evaluation with a larger, demographically representative consumer panel are also needed before broader application.

## Figures and Tables

**Table 1:** Phytochemical, antioxidant, proximate, and mineral composition of mango peel powder (MPP).

Parameter	MPP
Phytochemical and antioxidant properties	
Total carotenoids (mg/g dw)	1.31 ± 0.21
Total flavonoids (mg CE/g dw)	1.15 ± 0.25
Total polyphenols (mg GAE/g dw)	4.52 ± 0.29
Antioxidant activity (µmol TE/g dw)	1074.24 ± 1.64
ABTS radical inhibition (%)	81.23 ± 0.32
Proximate composition	
Ash (%)	2.48 ± 0.10
Moisture (%)	6.44 ± 0.14
Protein (%)	6.32 ± 0.12
Crude fiber (%)	32.58 ± 0.24
Lipids (%)	3.79 ± 0.11
Carbohydrates (%)	48.39 ± 0.35
Mineral composition	
Calcium (mg/100 g dw)	259.50 ± 0.38
Phosphorus (mg/100 g dw)	145.50 ± 0.19
Potassium (mg/100 g dw)	961.00 ± 0.36
Magnesium (mg/100 g dw)	118.25 ± 0.32
Zinc (mg/100 g dw)	0.76 ± 0.31
Iron (mg/100 g dw)	0.99 ± 0.20

Note: Values are expressed as mean ± standard deviation. MPP, mango peel powder; dw, dry weight; CE, catechin equivalents; GAE, gallic acid equivalents; TE, Trolox equivalents; ABTS, 2,2′-azino-bis(3-ethylbenzothiazoline-6-sulfonic acid). Proximate composition is expressed as a percentage of powder mass; mineral concentrations and the indicated phytochemical and antioxidant values are expressed on a dry-weight basis.

**Table 2:** Instrumental colour parameters of mango peel powder (MPP).

Parameter	MPP
L*	50.64 ± 0.13
a*	10.18 ± 0.12
b*	28.42 ± 0.06
C*	30.19 ± 0.08
h° (degrees)	70.29 ± 0.01

Note: Values are expressed as mean ± standard deviation. MPP, mango peel powder; L*, lightness; a*, red–green colour coordinate; b*, yellow–blue colour coordinate; C*, chroma; h°, hue angle.

**Table 3:** Physicochemical characteristics of the control and MPP-enriched cream cheese formulations.

Parameter	SC	MPPC1	MPPC2
Moisture, %	60.72 ± 0.07 ^a^	55.69 ± 0.11 ^b^	51.97 ± 0.06 ^c^
Fat, %	19.33 ± 0.09 ^b^	20.11 ± 0.07 ^a^	20.22 ± 0.04 ^a^
Protein, %	15.11 ± 0.12 ^b^	16.24 ± 0.13 ^ab^	16.85 ± 0.18 ^a^
Crude fiber, %	0.00 ± 0.00 ^c^	1.54 ± 0.08 ^b^	3.05 ± 0.10 ^a^
Carbohydrates, %	4.00 ± 0.06 ^c^	5.50 ± 0.02 ^b^	7.00 ± 0.08 ^a^
Ash, %	0.84 ± 0.05 ^a^	0.92 ± 0.07 ^a^	0.91 ± 0.08 ^a^
pH	5.88 ± 0.04 ^a^	5.74 ± 0.05 ^a^	5.63 ± 0.06 ^a^
Energy value, kcal/100 g	250.41 ± 0.21 ^c^	267.95 ± 0.23 ^b^	277.38 ± 0.24 ^a^
Energy value, kJ/100 g	1047.72 ± 0.88 ^c^	1121.10 ± 0.96 ^b^	1160.56 ± 1.00 ^a^

Note: Values are expressed as mean ± standard deviation from three independent production batches. SC, control spreadable cream cheese; MPPC1 and MPPC2, spreadable cream cheese prepared with nominal mango peel powder addition levels of 3 and 6 g/100 g of the curd–butter mixture before powder addition, respectively. Compositional percentages are expressed on a product-mass basis. Within each row, values sharing at least one lowercase superscript letter do not differ significantly, whereas values with no letters in common differ significantly (*p* < 0.05).

**Table 4:** Phytochemical characteristics and antioxidant activity of control and MPP-enriched cream cheese formulations during 21 days of refrigerated storage.

Parameter	Formulation	Day 0	Day 21	Retention, %
Total carotenoids, mg/g dw	SC	<LOQ	<LOQ	—
	MPPC1	0.38 ± 0.23 ^bA^	0.31 ± 0.20 ^bA^	81.58
	MPPC2	0.57 ± 0.26 ^aA^	0.49 ± 0.25 ^aA^	85.96
Total flavonoids, mg CE/g dw	SC	0.27 ± 0.15 ^cA^	0.20 ± 0.09 ^cA^	74.07
	MPPC1	0.46 ± 0.16 ^bA^	0.38 ± 0.11 ^bB^	82.61
	MPPC2	0.55 ± 0.18 ^aA^	0.47 ± 0.15 ^aB^	85.45
Total polyphenols, mg GAE/g dw	SC	0.85 ± 0.11 ^cA^	0.72 ± 0.10 ^cB^	84.71
	MPPC1	1.15 ± 0.15 ^bA^	1.01 ± 0.13 ^bB^	87.83
	MPPC2	1.66 ± 0.18 ^aA^	1.51 ± 0.14 ^aB^	90.96
Antioxidant activity, µmol TE/g dw	SC	505.20 ± 12.42 ^cA^	502.68 ± 11.36 ^cB^	99.50
	MPPC1	590.10 ± 15.25 ^bA^	587.88 ± 13.21 ^bB^	99.62
	MPPC2	672.21 ± 18.21 ^aA^	669.78 ± 14.58 ^aB^	99.64

Note: Values are expressed as mean ± standard deviation from three independent production batches. Retention (%) was calculated as (day 21 mean/day 0 mean) × 100. SC, control cream cheese; MPPC1 and MPPC2, cream cheese containing 3% and 6% mango peel powder, respectively; dw, dry weight; CE, catechin equivalents; GAE, gallic acid equivalents; TE, Trolox equivalents; LOQ., limit of quantification. For each parameter, different lowercase superscript letters indicate significant differences among the measured formulations on the same day (*p* < 0.05). Different uppercase superscript letters indicate significant differences between days 0 and 21 within the same formulation (*p* < 0.05). Values sharing the same letter within the relevant comparison do not differ significantly (*p* > 0.05).

**Table 5:** Instrumental colour parameters of control and MPP-enriched cream cheese during refrigerated storage.

Formulation	Storage Time (Days)	L*	a*	b*	C*	h°	ΔE
SC	0	90.04 ± 0.21 ^aA^	−2.19 ± 0.02 ^cB^	19.44 ± 0.10 ^cB^	19.56 ± 0.16 ^cB^	96.43 ± 0.01 ^aA^	—
SC	21	88.68 ± 0.18 ^aB^	−1.98 ± 0.01 ^cA^	20.32 ± 0.11 ^cA^	20.42 ± 0.20 ^cA^	95.57 ± 0.01 ^aB^	—
MPPC1	0	81.83 ± 0.20 ^bA^	2.62 ± 0.20 ^bB^	23.04 ± 0.03 ^bB^	23.19 ± 0.22 ^bB^	83.51 ± 0.01 ^bA^	10.17 ± 0.16 ^bB^
MPPC1	21	79.59 ± 0.17 ^bB^	3.02 ± 0.15 ^bA^	24.31 ± 0.10 ^bA^	24.50 ± 0.25 ^bA^	82.92 ± 0.01 ^bB^	11.12 ± 0.21 ^bA^
MPPC2	0	76.20 ± 0.16 ^cA^	4.53 ± 0.23 ^aB^	25.95 ± 0.02 ^aB^	26.34 ± 0.11 ^aB^	80.10 ± 0.01 ^cA^	16.71 ± 0.17 ^aB^
MPPC2	21	74.54 ± 0.15 ^cB^	5.11 ± 0.21 ^aA^	27.21 ± 0.01 ^aA^	27.69 ± 0.20 ^aA^	79.36 ± 0.01 ^cB^	17.26 ± 0.21 ^aA^

Note: Values are expressed as mean ± standard deviation from three independent production batches. SC, control cream cheese; MPPC1 and MPPC2, cream cheese containing 3% and 6% mango peel powder, respectively. L*, lightness; a*, red–green coordinate; b*, yellow–blue coordinate; C*, chroma; h°, hue angle (degrees); ΔE, total colour difference between an MPP-containing formulation and SC measured on the same storage day. For each colour parameter and storage day, different lowercase superscript letters indicate significant differences among formulations (*p* < 0.05). Different uppercase superscript letters indicate significant differences between days 0 and 21 within the same formulation (*p* < 0.05). Values sharing the same letter within the relevant comparison do not differ significantly (*p* > 0.05).

**Table 6:** Instrumental texture parameters of the control and MPP-enriched cream cheese formulations at day 0.

Parameter	SC	MPPC1	MPPC2
Hardness, N	4.38 ± 0.18 ^c^	5.59 ± 0.21 ^b^	7.09 ± 0.26 ^a^
Adhesiveness, mJ	0.46 ± 0.15 ^c^	0.70 ± 0.17 ^b^	0.94 ± 0.18 ^a^
Cohesiveness	0.39 ± 0.12 ^b^	0.50 ± 0.13 ^ab^	0.61 ± 0.17 ^a^
Springiness	0.41 ± 0.10 ^b^	0.52 ± 0.15 ^ab^	0.69 ± 0.20 ^a^
Gumminess, N	1.71 ± 0.23 ^c^	2.80 ± 0.26 ^b^	4.32 ± 0.28 ^a^
Chewiness, N	0.70 ± 0.17 ^c^	1.45 ± 0.19 ^b^	2.98 ± 0.29 ^a^

Note: Values are expressed as mean ± standard deviation from three independent production batches measured at day 0. SC, control cream cheese; MPPC1 and MPPC2, cream cheese containing 3% and 6% mango peel powder, respectively. Hardness, gumminess, and chewiness are expressed in newtons (N); adhesiveness is expressed in millijoules (mJ). Cohesiveness and springiness are dimensionless ratios. Within each row, formulations bearing different lowercase superscript letters differ significantly (*p* < 0.05), whereas formulations sharing at least one letter do not differ significantly (*p* > 0.05).

**Table 7:** Mineral composition of control and MPP-enriched cream cheese formulations.

Mineral, mg/100 g	SC	MPPC1	MPPC2
Calcium (Ca)	97.02 ± 0.34 ^c^	100.87 ± 0.36 ^b^	104.75 ± 0.37 ^a^
Phosphorus (P)	24.07 ± 0.24 ^c^	28.13 ± 0.25 ^b^	32.29 ± 0.27 ^a^
Potassium (K)	132.11 ± 0.30 ^c^	143.79 ± 0.33 ^b^	155.58 ± 0.36 ^a^
Magnesium (Mg)	9.56 ± 0.20 ^c^	13.70 ± 0.26 ^b^	18.39 ± 0.29 ^a^
Sodium (Na)	314 ± 0.17 ^a^	304.64 ± 0.19 ^b^	295.28 ± 0.25 ^c^
Zinc (Zn)	0.50 ± 0.06 ^b^	0.54 ± 0.08 ^ab^	0.57 ± 0.10 ^a^

Note: Mineral concentrations are expressed as mg/100 g of cheese and reported as mean ± standard deviation from three independent production batches. SC, control cream cheese; MPPC1 and MPPC2, cream cheese containing 3% and 6% mango peel powder, respectively. Within each row, formulations with different lowercase superscript letters differ significantly (*p* < 0.05), whereas formulations sharing at least one letter do not differ significantly (*p* > 0.05).

## Data Availability

The original contributions presented in the study are included in the article, further inquiries can be directed to the corresponding author.
